# Inverted Silicon Nanopencil Array Solar Cells with Enhanced Contact Structures

**DOI:** 10.1038/srep34139

**Published:** 2016-09-27

**Authors:** Xiaoguang Liang, Lei Shu, Hao Lin, Ming Fang, Heng Zhang, Guofa Dong, SenPo Yip, Fei Xiu, Johnny C. Ho

**Affiliations:** 1 Department of Physics and Materials Science, City University of Hong Kong, 83 Tat Chee Avenue, Kowloon Tong, Kowloon, Hong Kong; 2 Shenzhen Research Institute, City University of Hong Kong, 518057 Shenzhen, P. R. China; 3 Key Laboratory of Flexible Electronics (KLOFE) & Institute of Advanced Materials (IAM), Jiangsu National Synergetic Innovation Center for Advanced Materials (SICAM), Nanjing Tech University (NanjingTech), 30 South Puzhu Road, Nanjing 211816, P. R. China; 4 State Key Laboratory of Millimeter Waves, City University of Hong Kong, 83 Tat Chee Avenue, Kowloon Tong, Kowloon, Hong Kong; 5 Centre for Functional Photonics (CFP), City University of Hong Kong, 83 Tat Chee Avenue, Kowloon Tong, Kowloon, Hong Kong

## Abstract

Although three-dimensional nanostructured solar cells have attracted extensive research attention due to their superior broadband and omnidirectional light-harvesting properties, majority of them are still suffered from complicated fabrication processes as well as disappointed photovoltaic performances. Here, we employed our newly-developed, low-cost and simple wet anisotropic etching to fabricate hierarchical silicon nanostructured arrays with different solar cell contact design, followed by systematic investigations of their photovoltaic characteristics. Specifically, nano-arrays with the tapered tips (e.g. inverted nanopencils) are found to enable the more conformal top electrode deposition directly onto the nanostructures for better series and shunt conductance, but its insufficient film coverage at the basal plane would still restrict the charge carrier collection. In contrast, the low-platform contact design facilitates a substantial photovoltaic device performance enhancement of ~24%, as compared to the one of conventional top electrode design, due to the shortened current path and improved lateral conductance for the minimized carrier recombination and series resistance. This enhanced contact structure can not only maintain excellent photon-trapping behaviors of nanostructures, but also help to eliminate adverse impacts of these tapered nano-morphological features on the contact resistance, providing further insight into design consideration in optimizing the contact geometry for high-performance nanostructured photovoltaic devices.

In the past decade, due to their unique physical properties such the low reflectance and efficient photon harvesting, three-dimensional (3-D) silicon (Si) nanostructures such as the nanowire, nanopillar and nanocone arrays have not only attracted extensive attentions for solar cells, but also inducing significant impacts in various sensor and transistor technologies^1^,^2^,^3^,^4^,^5^,^6^,^7^,^8^. Especially for photovoltaics, in order to achieve nanostructures with high aspect ratios, reactive ion etching (RIE)^9^,^10^. electron beam lithography^11^,^12^ (EBL) and metal-assisted chemical etching (MaCE) approaches^13^,^14^,^15^ were widely exploited for the construction of solar cell devices. In any case, all these methods would intrinsically lead to the high processing cost with low throughput and at the same time inevitably roughen the fabricated Si surfaces deteriorating their resulting photovoltaic performances^4^,^7^,^15^. Recently, we have developed an unique large-scale, low-cost and nanoscale patterning technique employing soft polymer photomasks as well as a simple but reliable wet-chemistry-only anisotropic etching scheme to attain Si nano-arrays with different geometrical morphologies, including inverted nanopencil, nanopillar and nanocone arrays^16^,^17^,^18^. Both experimental and simulated results have indicated that these as-made nanostructured window layers, particularly the inverted nanopencil arrays, possess excellent broadband and omnidirectional light harvesting properties^19^; however, such nano-surface textured crystalline Si solar cells are still suffered from the disappointed power conversion efficiency (PCE), although they have demonstrated the remarkable light trapping properties^20^. For example, the non-conformal metal mesa bar contact (i.e. top or front electrodes) induced by the fluctuated surface and very short pitch of nano-arrays would yield the substantial parasitic resistance, and thus aggravate the photo-generated carrier collection and degrade the overall conversion efficiency^21^,^22^. This way, several enhanced photovoltaic contact structures have been proposed and reported, comprising the heavily doped contact region and such, which aim to minimize the series and shunt resistance^23^,^24^. But the heavy doping in Si (i.e. doping concentration >1 × 10^18^ cm^3^) would inexorably make the Auger recombination being the dominant recombination process, giving the low lifetime and short diffusion length of minority carriers^25^,^26^,^27^. On the other hand, the design of nanostructured solar cells can also be manipulated in a way that the nanostructured window layer and the metal mesa bar contact with small area are spatially separated to alleviate the negative impact of these nanostructures on their electrical contact properties, without disturbing their excellent broadband and omnidirectional light absorption characteristics^28^,^29^; nonetheless, there is limited work focusing on these improved contact schemes to date. In this regard, optimizing the solar cell design to effectively combine the nanostructured window, mesa bar contact and high-quality junction could be an important step towards the practical utilization of nanostructured photovoltaics operated with the ultimate photovoltaic performance limit.

In the present study, we explore the use of our newly-developed, low-cost and simple wet anisotropic etching technique to fabricate hierarchical Si nanostructured arrays^16^, ranging from nanopillars, nanocones all the way to inverted nanopencils, with separated mesa bar contact regions^23^, followed by systematic investigations of their photovoltaic performances. Specifically, three different contact structures are emphasized here, involving (1) the contact directly fabricated on nanostructures, (2) the mesa bar contact and (3) the low-platform contact. It is found that nano-arrays with the tapered tips, that are nanocones and inverted nanopencils, would enable the more conformal formation of top electrode contact directly onto the nanostructures for the better series and shunt conductance, regardless of the deposition techniques utilized. Moreover, solar cells with the low-platform contact is found to facilitate the significant photovoltaic device performance enhancement of ~24%, as compared to the one of conventional top electrode contact design due to the shortened current path and improved lateral conductance for the minimized carrier recombination (i.e. ~18% improvement in short-current current density) and shunt resistance (i.e. ~8% reduction in series resistance). This exploration illustrates a comprehensive investigation of developing low-platform contact for the enhanced nanostructured solar cells, offering not only the excellent omnidirectional light absorption, but also the efficient carrier separation and collection. More importantly, all these would provide essential insight into the design consideration in optimizing the contact geometry for the high-performance nanostructured photovoltaic devices.

## Results

### Conformal coating of the top electrode

**Figure 1:**
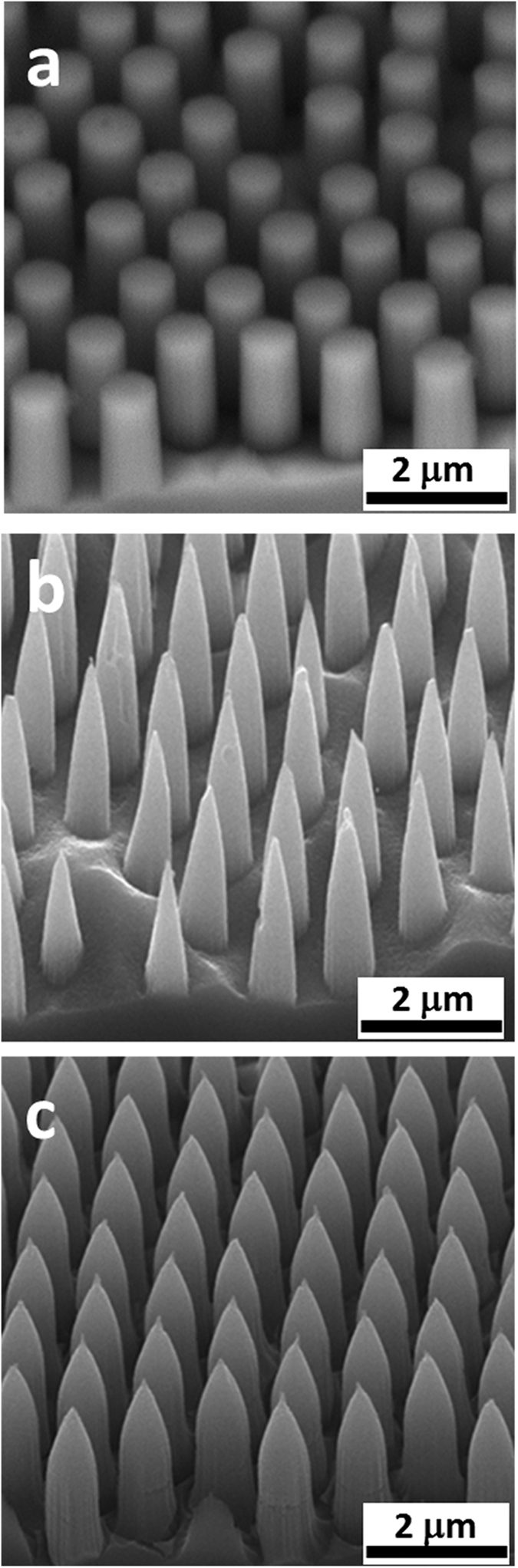
45° angle-view SEM images of silicon nanopillar (**a**), nanocone (**b**) and nanopencil (**c**) arrays obtained by the wet anisotropic etching. The structural pitch, pillar base diameter and height are 1.27 μm, 0.6 μm and 2 μm, respectively. The tip height and angle are controlled to be around 1 μm and 45° for the nanopencil, accordingly.

**Figure 2:**
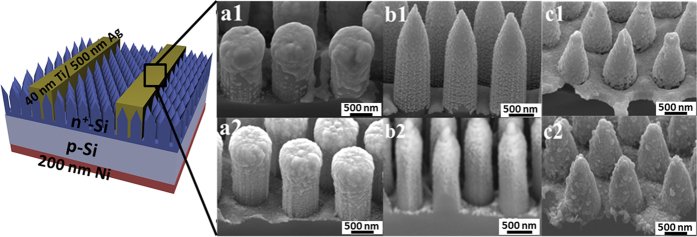
Schematic illustration of silicon nanostructure arrayed solar devices and corresponding 45° angle-view SEM images of the top electrode contact (Ti/Ag) directly deposited on nanopillars, inverted nanopencils, nanocones by evaporator (a1–c1) and by magnetron sputtering (a2–c2), respectively. The schematic is not drawn in scale.

Figure 1 demonstrates the scanning electron microscope (SEM) images of regular Si nanopillar, nanocone and inverted nanopencil arrays fabricated with the controllable structural pitch, diameter, height, aspect ratio (i.e. pillar height-to-base diameter) and material-filling ratio (i.e. base diameter-to-pitch), in which their large-scale fabrication can be reliably obtained by simply manipulating the dimension of polystyrene spheres (PS) during the nanoscale patterning, the concentration of etchant solution and the processing duration as previously reported^19^. Afterwards, in fabrications of most nanostructured based solar cells, after the high-quality p-n junction is formed, the top electrode is usually deposited directly onto the nanostructures, and the uniformity of this electrode coverage is essential for the improved photovoltaic device performance. In order to qualitatively assess the conformality of the deposited top electrode, various conventional thin film deposition techniques, namely the evaporation and sputtering, are employed to coat ~540 nm thick of the typical metal electrode stack (40 nm Ti/ 500 nm Ag) directly onto the nano-arrays with the pitch of ~1.2 μm and the height of ~2 μm. As shown in the SEM image in Fig. 2, it is anticipated that the evaporation technique would give non-conformal coatings to all nanostructured arrays due to its line-of-sight deposition process. Although the substrate rotation is always utilized to enhance the evaporated film coverage for the outer surface of these complex geometries, it is still not capable to coat the inner surface of such structures, especially the basal plane of the nano-arrays studied in this work (Fig. 2a1–c1). On the contrary, the sputtering approach can yield the more conformal coating on most nano-arrays since the sputtered atoms ejected into the plasma are existed in their thermodynamic non-equilibrium states with enhanced mobility to result in the scattered deposition for the better film coverage (Fig. 2a2–c2). In addition to the influence of different deposition techniques, the geometrical morphology of nano-arrays is also observed to have a great impact on the uniformity of the deposited electrode stack. It is clear that when the material-filling ratio is kept constant along the height of the array (i.e. nanopillars), this limited open space would restrict the movement of metal particles towards the basal plane during the deposition and eventually lead to the preferential accumulation of particles at the tip area to form the mushroom-like feature (Fig. 2a1,a2). For the arrays with tapered tips (i.e. nanocones and inverted nanopencils), there are more capture cross-section areas available at the tip region, allowing the passage of metal particles to reach the basal plane in a more continuous and compact manner. It should also be noted that the typical p-n junction formation processed by spin-on dopants (SODs) or gas diffusion can as well be easily and uniformly achieved in these tapered nano-arrays due to the similar reason (Supporting Information Figure S1). More importantly, the advantage of using these tip-tapered nano-arrays in attaining the conformal coating as well as the continuous junction formation is even more profound for arrays with the smaller structural pitch and higher aspect ratio, indicating the potency of these nanocones and nanopencils for the highly efficient solar cell structure with the conformal front electrodes directly deposited on the top and the formation of high-quality junctions.

### Photovoltaic device performances

**Figure 3:**
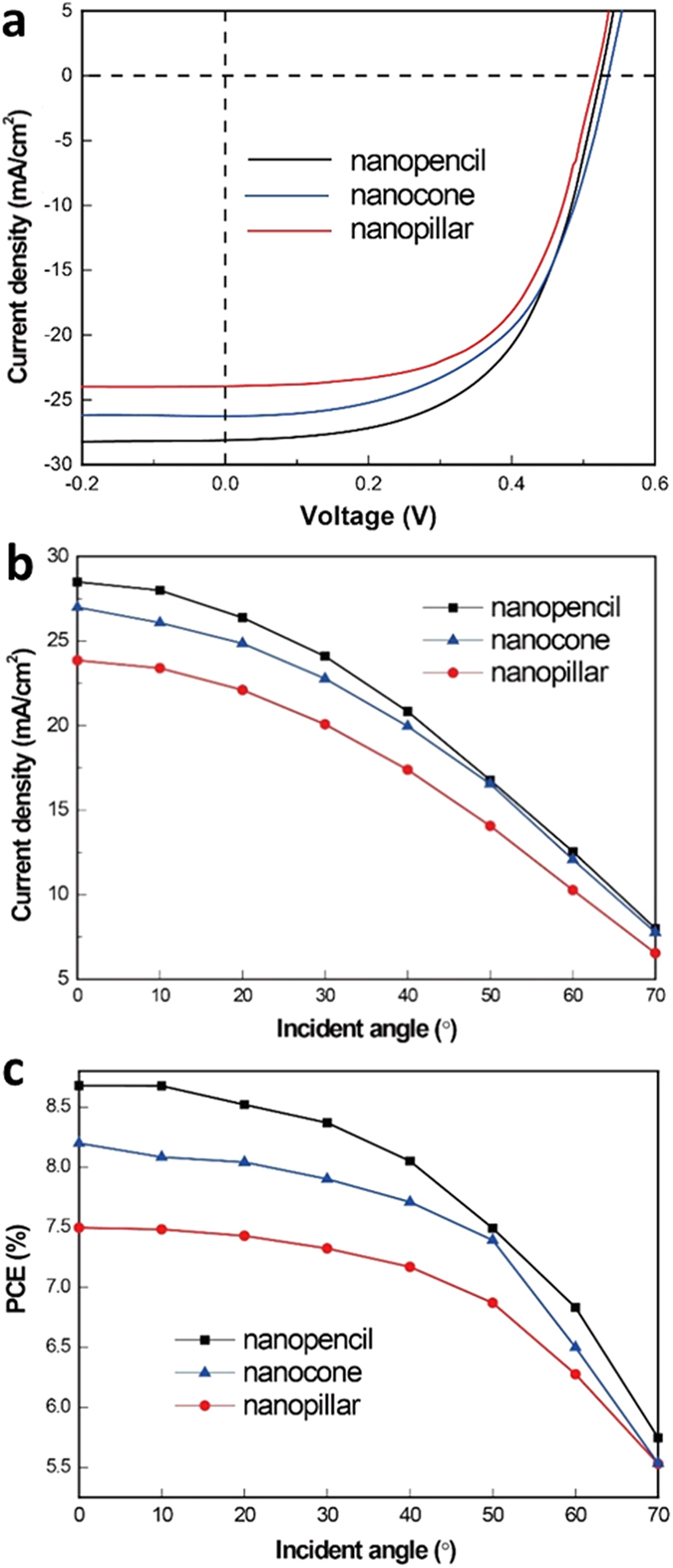
(**a**) Current density-voltage (J-V) curves of Si nanostructure arrayed solar cells with top electrode contact directly deposited on the top. (**b**) The corresponding angle-dependent short-circuit current density and (**c**) photovoltaic conversion efficiency of these Si nanostructured devices.

Once the conformality of the top electrode stack is assessed, it is important to evaluate their corresponding effect on the photovoltaic device performance of all nano-arrayed solar cell devices. Notably, even though these solar devices are not optimized for their best efficiency, their geometrical parameters, except for the morphology, and fabrication conditions (e.g. sputtered top electrodes) are maintained the same for all samples studied in this work for the fair comparison. Figure 3a illustrates the photovoltaic properties of all different types of nano-arrayed cells measured under AM 1.5 G (i.e. 1000 W/m^2^ at 25 °C) at the normal incident angle. Obviously, all these devices display the similar open-circuit voltage (*V*_*oc*_) of ~0.53 V and fill factors (FF) of ~0.58 but with the remarkable difference in the short-circuit current (*J*_*sc*_) and PCE. In specific, the nanopencil arrayed cell exhibits the highest *J*_*sc*_ of 28.5 mA/cm^2^ and PCE of 8.7% among all devices. Furthermore, as shown in Fig. 3b,c, the nanopencil cell also demonstrates the excellent omnidirectional characteristics, maintaining over 95% and 80% of their PCEs for the incident angle up to 40° and 60°, respectively, which has nearly 20% of improvement in PCE and *J*_*sc*_ over the conventional nanopillar devices. All these significant enhancements can be attributed to the excellent light absorption characteristics for the effective photo-carrier generation as well as the better top electrode coverage directly deposited on the tip-tapered nanostructures for the efficient carrier collection as discussed above. Based on the previous optical study^19^, although the nanopencil arrays have been revealed to suppress the optical reflection well below 5% over the solar spectrum and the wide angle of incidence between 0° and 60°, the same performance in PCE and *J*_*sc*_ are not observed for the fabricated solar devices here, suggesting that the top electrode coverage in the basal plane of nano-arrays is still not compact and continuous enough for the minimization of contact resistance. Importantly, the increased interfacial area of the contact region could badly deteriorate the charge collection efficiency of these 3-D nanostructure-based solar devices due to the significant surface/interface recombination^27^,^30^. In this case, in order to further depress and eliminate the undesired recombination effect, an alternative design on the contact structure is essentially required for the devices.

### Enhanced contact structures

**Figure 4:**
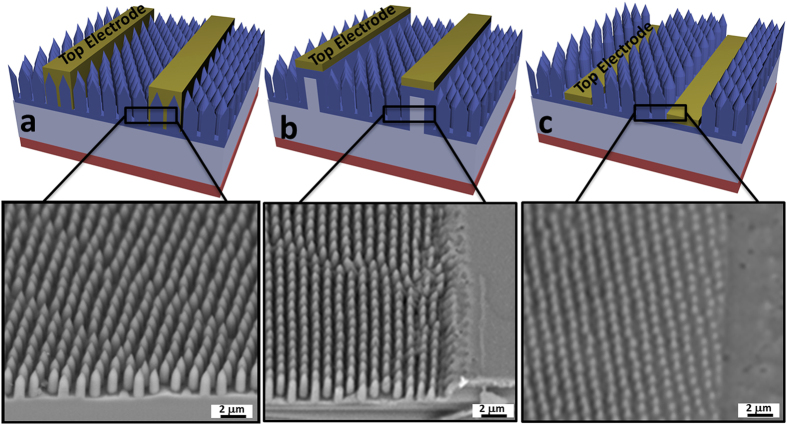
Schematic illustration and 45° angle-view SEM images of different top electrode contact design with (**a**) metal directly deposited on top of the structure, (**b**) mesa bar metal strips and (**c**) low-platform metal strips before the metal stack deposition. It is noted that nanopencil arrays are employed here due to their observed superior light absorption characteristics for the improved PCE of fabricated solar devices. Schematics are not drawn in scale.

With the aim to enhance the charge collection efficiency, we purposely propose and fabricate several enhanced contact structures, which can spatially separate the nanostructured window layer and mesa bar contact with small area such that the photo-carrier generation and collection are decoupled from each other. Specifically, by combining MaCE, nanosphere lithography and conventional photolithography, hierarchically arranged Si nanopecil arrays can be achieved as depicted in Fig. 4 23. Nanopencil arrays are exploited here due to their observed superior light absorption characteristics for the improved PCE of fabricated solar devices. Figure 4a shows the SEM image of the fabricated nanopencil arrays while Fig. 4b,c display the top electrode region of the mesa bar and low-platform contact structures, respectively, before the metal stack deposition. It is noticed that these hierarchically arranged nanopencil arrays fabricated for the mesa bar and low-platform contacts can be readily obtained by changing the sequence of lithographic processes in order to achieve the site-selective etching and fabrication of nanostructured arrays^23^. For instance, before the nanosphere lithography, the mesa bar feature could be first pre-patterned onto the silicon surface by conventional photolithographic, sputtering and lift-off processes; this way, after the catalyst deposition and anisotropic wet etching were performed, the nanopencil arrays as well as the mesa bar contact structure could be attained simultaneously for the solar cell fabrication (Fig. 4b). Similarly, to achieve the low-platform contact scheme, the patterned catalyst nanomesh was instead first patterned onto the silicon surface by nanosphere lithography, followed by Au deposition on the defined contact region served as catalysts for the subsequent etching (Fig. 4c).

**Figure 5:**
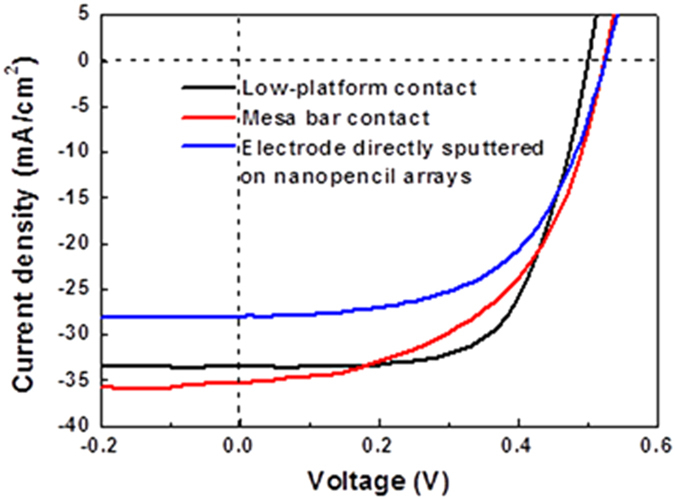
Current density-voltage (J-V) curves of inverted Si nanopencil arrayed solar cells with different top electrode contact design: directly deposited on top of the nanopencil arrays, mesa bar and low-platform configuration.

Figure 5 illustrates the corresponding photovoltaic performance of inverted nanopencil arrays based solar cell devices with these three different top electrode contact structures, with Table 1 summarized the important device parameters. Similar open-circuit voltage (*V*_*oc*_) values are observed for all these devices since they are processed with the same doping condition and silicon starting substrates for the uniform junction formation. It is obvious that although these tip-tapered nanopencil arrays would lead to a more conformal and continuous top electrode formation, there is still a substantial film coverage issue existed in the basal plane, which is further confirmed with the corresponding top-view optical image (Supporting Information Figure S2), resulting in the inferior charge carrier collection and hence the lowest *J*_*sc*_ of 28.5 mA/cm^2^ among all contact schemes. Once the nanostructured window layer is separated from the contact region, the *J*_*sc*_ of both mesa bar and low-platform contacts are improved significantly by at least a 20% of enhancement. As compared to the *J*_*sc*_ of 35.3 mA/cm^2^ of the mesa bar contact, a slightly lower *J*_*sc*_ of 33.6 mA/cm^2^ of the low-platform contact is obtained, which can be attributed to the severe carrier recombination due to the relatively rough top electrode/silicon interface induced by the device fabrication. This roughness is mainly come from the formation of small cracks between the two deposited metal layers at the hole edges of the nanomesh and the subsequent exposure in the etching solution^23^. However, the mesa bar contact scheme would inevitably increase the associated series resistance to 3.1 ohm-cm^2^ as contrasted to the one of 2.3 ohm-cm^2^ of the low-platform contact because of its longer current path required, approximating the height of the nanopencil, and thus impair its corresponding FF and PCE of the fabricated devices. This longer current path issue is expected to even get worsen for the taller features. In this case, owing to the shortened current path and good lateral conductance, the efficient carrier collection of *J*_*sc*_ being 33.6 mA/cm^2^ and low series resistance of 2.2 ohm-cm^2^ are readily achieved for the low-platform contacted device. More importantly, this enhanced contact design can prevent potential shunts from the nanostructured surface and result in a high FF of 0.64, which leads to the highest PCE of 10.8% performed in this work. All these have elucidated the importance of contact design consideration of highly-efficient nanostructure-based solar devices for the optimization of light absorption, carrier collection, leakage elimination and lateral conductance.

## Discussion

In recent years, although three-dimensional nanostructured solar cells have attracted extensive research attention due to their superior broadband and omnidirectional light harvesting properties, majority of these devices are suffered from complicated fabrication processes as well as disappointed photovoltaic performances. One of the key challenges is to optimize the cell design combining the nanostructured window, enhanced electrode contact and high-quality junction effectively towards their practical deployment with the ultimate performance limit. The current work adopts a simple and low-cost wet anisotropic etching to fabricate various silicon nano-arrays, including nanopillars, nanocones and inverted nanopencils, followed by the investigation of their photovoltaic performances with different solar device contact structures. Importantly, the nanocone and nanopencil arrays would result in the more conformal and uniform top electrode contact directly deposited on top of the nanostructures, regardless of the deposition techniques employed; however, there is still significant film coverage issue at the nano-array basal plane, which restricts the corresponding charge carrier collection and thus the power conversion efficiency. At the same time, the enhanced contact designs can also be implemented to spatially separate the nanostructured window layer and the contact electrode in order to decouple the required photo-generation and collection. Among all the structures studied, the low-platform contact scheme is observed to yield the best photovoltaic performance of nanopencil arrayed cells with the power conversion efficiency of 10.8% due to the shortened current path and improved lateral conduction for the minimized carrier recombination and series resistance. As a distinct contrast to the conventional top electrode contact design, this low-platform contact scheme can facilitate a significant improvement in *J*_*sc*_ of ~18% and an impressive reduction in series resistance of ~8%. This way, the superior light-harvesting properties of nanostructured arrays can be maintained while the negative effect of these arrays on the electrical contact characteristics can also be minimized. All these findings would provide valuable insight into the design consideration in optimizing the contact geometry for the high-performance nanostructured photovoltaic devices.

## Methods

### Fabrication of different nanostructured arrays

P-type <100>-oriented Czochralski (CZ) Si wafers with a resistivity of 1–10 ohm-cm and a thickness of 500 μm were used as the starting substrate to fabricate 3D nanostructures and site-selective electrode contact structures via an anisotropic wet etching technique as reported before^16^,^23^. In brief, monodispersed polystyrene (PS) nanospheres with different controllable diameters were assembled into a close-packed monolayer on the substrates using the Langmuir–Blodgett (LB) method. The substrates were pre-treated with mild oxygen plasma to induce hydrophilic surfaces for the facilitation of uniform nanosphere coating. The diameter and spacing of the spheres can be further manipulated by subsequent oxygen plasma etching. Using these spheres as the mask (i.e. nanosphere lithography), a 1.5/20 nm thick Ti/Au nanomeshed film could be deposited through thermal evaporation and Si nanopillars with an adjustable diameter and structural pitch could be achieved via MaCE in HF/H_2_O_2_ solution^16^. These nanopillar arrays were next used as fabrication templates for the construction of nanocone arrays and inverted nanopencil arrays. Once the templates were attained, they were treated with a mixture of AgNO_3_, HF, and HNO_3_ or H_2_O_2_. By modulating the ratio and components of the etching mixture, different etching reaction kinetics can be obtained to yield different morphological features such as nanocones and inverted nanopencils^16^.

### Fabrication of nanostructured based photovoltaic devices

When the nanostructured arrays were obtained, all samples were spin-coated and chemically doped with the phosphorous-containing spin-on-dopant (SOD) to achieve a n^+^/p junction under an annealing condition of 950 °C for 50 s by using rapid thermal annealing (RTA), following by the reactive-ion etching (RIE) performed at the back-side in order to eliminate the n-type layer there. Secondary ion mass spectrometry (SIMS) was then carried out to assess the corresponding phosphorous-doped junction depth. Since the determined junction depth of ~400 nm is greater than the radius of nanopillar, it is confirmed that the planar n^+^/p junction is formed underneath the nanostructured arrays (Supporting Information Figure S3). Magnetron sputtering or electron-beam evaporation was utilized to deposit the top metal electrode contact (40 nm Ti/500 nm Ag) with the electrode pattern pre-defined by the conventional lithography, followed by the lift-off process (Supporting Information Figure S2). The bottom electrode (200 nm Ni) was then deposited by thermal evaporation. After the deposition of metal contacts on both sides, the samples were cut off into small cells for subsequent studies with an area of 20 × 20 mm^2^. These devices were finally annealed at 400 °C for 10 min under H_2_ atmosphere for the formation of ohmic-like contact.

For mesa bar and low-platform contacted cells, additional photolithographic steps are utilized in the device fabrication to achieve the site-selective anisotropic wet etching to spatially separate the nanostructured window for the efficient photon absorption and the top electrode contact for the effective charge carrier collection. In the mesa bar contact scheme, before the nanosphere monolayer is deposited onto the substrate, the mesa bar feature is first pre-patterned by the conventional photolithographic, sputtering and lift-off processes; as a result, after the catalyst is deposited, the patterned nanomesh feature is already pre-defined on the substrate surface for the subsequent anisotropic wet etching. On the contrary, in the low-platform contact structure, nanosphere lithography was used to obtain a 1.5/20 nm thick Ti/Au nanomeshed film at the first step. The as-made regular nanomesh pattern was then covered through a shadow mask, and subsequently another 20 nm thick Au was then deposited on the gaps of mask as catalysts on the top electrode^23^. Finally, the low-platform contact and hierarchical nanostructured arrays were achieved at the same time during the anisotropic wet etching process.

### Characterization of fabricated solar devices

Current-voltage (I-V) curves and angle-dependent photovoltaic performances were measured under AM 1.5 G (i.e. 1000 W/m^2^ at 25 °C) illumination by a solar simulator (Newport Oriel Instruments). Surface morphologies of different Si nano-textured arrays and different electrode contact structures were examined with a scanning electron microscope (SEM, Phenom Pro).

## Additional Information

**How to cite this article**: Liang, X. *et al.* Inverted Silicon Nanopencil Array Solar Cells with Enhanced Contact Structures. *Sci. Rep.* **6**, 34139; doi: 10.1038/srep34139 (2016).

## Supplementary Material

Supplementary Information

## Figures and Tables

**Table 1:** Electrical parameters of silicon nanopencil based solar cells with different top contact structures.

Contact Design	J_SC_ (mA/cm^2^)	V_OC_ (V)	FF	PCE (%)	R_S_ (ohm-cm^2^)
Electrode directly sputtered on nanopencil arrays	28.5	0.53	0.57	8.7	2.5
Mesa bar contact	35.3	0.52	0.53	9.6	3.1
Low-platform contact	33.6	0.50	0.64	10.8	2.3
