# Supplementary material for: Inverted Silicon Nanopencil Array Solar Cells with Enhanced Contact Structures
Source: Sci Rep. 2016 Sep 27;6:34139. doi: 10.1038/srep34139 (PMC5037459; doi:10.1038/srep34139)
Supplement: Supplementary Information [file srep34139-s1.pdf]

Supporting Information

Inverted Silicon Nanopencil Array Solar Cells with Enhanced Contact Structures

Xiaoguang Liang[1,2,†], Lei Shu[1,2†], Hao Lin[1,†], Ming Fang[1,2], Heng Zhang[1], Guofa Dong[1,2], SenPo Yip[1,2], Fei Xiu[3,*], Johnny C. Ho[1,2,4,5,*]

[1]Department of Physics and Materials Science, [4]State Key Laboratory of Millimeter Waves and [5]Centre for Functional Photonics (CFP), City University of Hong Kong, 83 Tat Chee Avenue, Kowloon Tong, Kowloon, Hong Kong

[2]Shenzhen Research Institute, City University of Hong Kong, 518057 Shenzhen, P.R. China

[3]Key Laboratory of Flexible Electronics (KLOFE) & Institute of Advanced Materials (IAM), Jiangsu National Synergetic Innovation Center for Advanced Materials (SICAM), Nanjing Tech University (NanjingTech), 30 South Puzhu Road, Nanjing 211816, P.R. China

*E-mail: Johnny C. Ho (johnnyho@cityu.edu.hk) & Fei Xiu (iamfxiu@njtech.edu.cn)

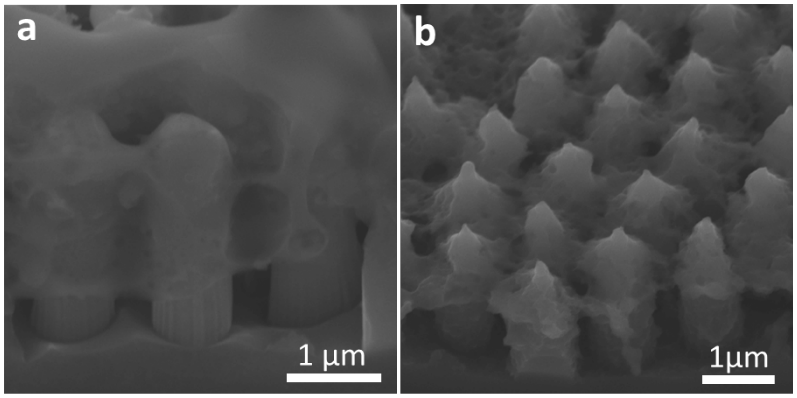

Figure S1. 45° angle-view SEM images of phosphorus spin-on dopants (P-SODs) spin-coated on the surface of nanopillars (a) and nanopencils (b) after the soft baking.

Figure S1a and b depict the SEM images of P-SODs spin-coated onto the nanopencil and nanopillar arrays, respectively, after the soft baking at 200 °C for 1 min. As indicated by Figure S1a, since the material filling ratio is constant along the height of the pillar, it is difficult for the SOD source to flow into the basal plane of the structure and therefore the nonhomogeneous distribution of the source is resulted. In contrast, as shown in Figure S1b, since the tip-tapered nanopencil has a gradual increase of the material filling ratio towards the basal plane of the structure, the SOD source can get into the bottom of the pillar easily and achieve the more conformal deposition of the source, facilitating the formation of homogeneous p/n junction there.

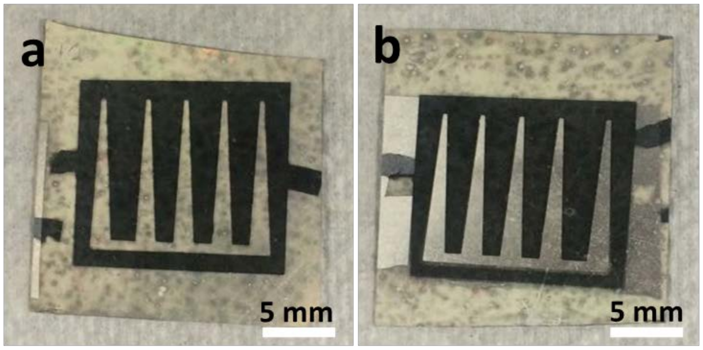

Figure S2. The top-view optical images of solar cell devices fabricated with the (a) top electrode directly deposited onto the nanopencils and (b) low-platform contact design.

Figure S2a demonstrates the rough and discontinuous top metal electrode (40 nm Ti/ 500 nm Ag) deposition directly on the nanopencil arrays while Figure S2b illustrates the dense and smooth top metal electrode (40 nm Ti/ 500 nm Ag) deposited for the low-platform contact scheme.

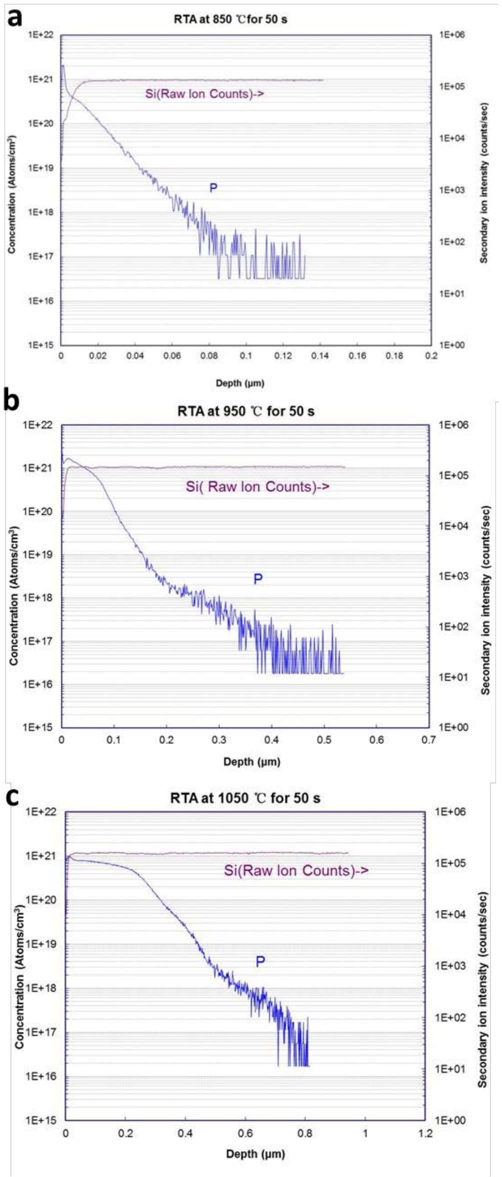

Figure S3. Secondary ion mass spectrometry (SIMS) of planar silicon substrate doped with phosphorus-SOD process by using rapid thermal annealing (RTA) at 850 °C (a) , 950 °C (b) and 1050 °C (c) for 50 s, respectively.

In this work, planar substrates are employed for the SIMS measurement in order estimate the junction depth obtained in the nanostructured arrays fabricated. Based on the RTA condition of 950 ^{o}C/50 s utilized in the doping process during the solar cell fabrication, a junction depth of around 400 nm is obtained. Since the pillar diameter is 0.6 µm, the dopant would diffuse into the bulk of the nanostructure to achieve a planar n^{+}/p junction below the nano-surface texture for all solar cell devices here.
